# Effect of statins and testosterone replacement therapy on incident cardiovascular disease among male hormone-related cancer survivors

**DOI:** 10.21203/rs.3.rs-3530181/v1

**Published:** 2023-11-03

**Authors:** Danielle El Haddad, Hyunkyoung Kim, Efstathia Polychronopolou, Jacques Baillargeon, Alejandro Villasante-Tezanos, Yong-Fang Kuo, Syed Gilani, Wissam I. Khalife, David S. Lopez

**Affiliations:** Internal Medicine- University of Texas Medical Branch; University of Texas Medical Branch; University of Texas Medical Branch; University of Texas Medical Branch; University of Texas Medical Branch; University of Texas Medical Branch; Internal Medicine- University of Texas Medical Branch; Internal Medicine- University of Texas Medical Branch; University of Texas Medical Branch

**Keywords:** statins, testosterone and cardiovascular disease, cancer, HRCa, TTh, statins, CVD

## Abstract

**Purpose:**

Statins and testosterone replacement therapy (TTh) have been previously linked with prostate, colorectal and male breast cancer (hereinafter we will refer as hormone related cancers [HRCa]), and cardiovascular disease (CVD). However, there is a poor understanding about the combined association of statins and TTh with incident CVD among HRCa survivors and a matched cancer-free cohort.

**Methods:**

We identified 44,330 men of whom 22,165 were previously diagnosed with HRCa, and 22,165 were age-and index-matched cancer-free in SEER-Medicare 2007–2015. Pre-diagnostic prescription of statins and TTh prior to CVD development was ascertained for this analysis in the two matched cohorts. Weighted multivariable-adjusted conditional logistic regression models were used to evaluate the independent and combined associations of statins and TTh with CVD.

**Results:**

We found that use of statins (OR = 0.51, 95% CI: 0.46–0.55) and TTh (OR = 0.81, 95% CI: 0.67–0.97) were each independently inversely associated with incident CVD in the overall sample. TTh plus statins was also inversely associated with CVD. Associations were similar in the matched cancer-free cohort. Among HRCa survivors, only statins and combination of TTh plus statins (OR = 0.60, 95% CI: 0.44–0.98) were inversely associated with CVD, but the independent use of TTh was not associated with CVD.

**Conclusion:**

In general, pre-diagnostic use of statins and TTh, prior to CVD development, independently or in combination, were inversely associated with CVD in the overall, cancer-free population, and among HRCa survivors (mainly combination). Independent effects and combination of statins and TTh remained to be confirmed with specific CVD outcomes among HRCa survivors.

## Introduction

The effect of statins on the reduction of overall CVD risk has been well studied, but it merits a further observation with prostate, colorectal and male breast cancers, and their combination, referred as hormone-related cancers (HRCa), with and without testosterone replacement therapy (TTh).^1–6^

Colorectal cancer is investigated as a testosterone-associated cancer and not as a hormone-dependent cancer or hormone-caused cancer; therefore, this potential testosterone-cancer relationship has potential translational applications for preventive diagnosis and risk stratification.^7–10^ In colorectal cancer research, a meta-analysis reported that statin use was a protective factor for colorectal cancer prognosis,^4^ but an umbrella systematic review and meta-analysis concluded that the evidence supporting the use of statins to reduce cancer mortality or survival was low.^5^ In prostate cancer research, a meta-analysis found an inverse association between use of statins and prostate cancer mortality.^11,12^A meta-analysis of 28 randomized controlled trials (RCTs) reported that statins therapy had no effect on overall cancer incidence, or cancer death.^2^

In parallel, the association of TTh with prostate, colorectal, male breast cancers and HRCa in large studies remains poorly understood.^6–9,13–15^ RCTs represent the most rigorous study design in terms of reducing confounding and selection bias, but they often have small samples with limited generalizability. A meta-analysis of 11 RCTs based on 20 incident prostate cancer cases concluded that testosterone supplementation for symptomatic hypogonadism did not increase the risk of prostate cancer^16^, but the power to make conclusions was low. Recent observational studies have linked serum testosterone levels with colorectal cancer^7–10,17^ and breast cancer in men^14,15^, but to date no RCT has examined these associations.

Cardiovascular diseases (CVD), remain the leading cause of death in the United States (U.S.), and represent an increasing concern for cancer survivors given prolongation of life expectancy with new advances in therapy.^18^ The recent rate of CVD among cancer survivors is increasing and is higher than in the general population^19^, which may be due, in part, to the cardiotoxicity of some cancer treatments. ^20^ An Evidence Report and Systematic Review from the US Preventive Services Task Force (19 randomized trials) reported an inverse association between statins and CVD.^21^ Yet, the association of TTh with CVD in larger studies remains unclear.^21^ In late 2013 and early 2014, two studies reported increased myocardial infarction and stroke associated with TTh use^22,23^; however, other studies investigating the relationship between endogenous/exogenous testosterone and CVD in systematic reviews and meta-analysis of observational studies and randomized controlled trials found inconclusive results.^24^ Of interest, there is a potential biological plausibility in the interaction between cholesterol and testosterone, and with their treatments, statins and TTh, because cholesterol is a required intermediate precursor in steroidogenesis.^25–27^ Therefore, the objective of this study is to investigate the independent and joint associations of statins and TTh with CVD among HRCa survivors and a matched cancer-free cohort.

## Methods

### Data Source

The study used Surveillance, Epidemiology and End Results (SEER)-Medicare-linked database from January 2007 to September 2015. The linked SEER Medicare files provide demographic and clinical information from population-based cancer registries of the Medicare program from 19 states in the US. We used the SEER Medicare data for hormone related cancer (prostate, colorectal and male breast) survivor populations and a 5% random sample of non-cancer Medicare beneficiary for non-cancer survivor populations in a SEER region.

### Study cohort

**Figure 1:**
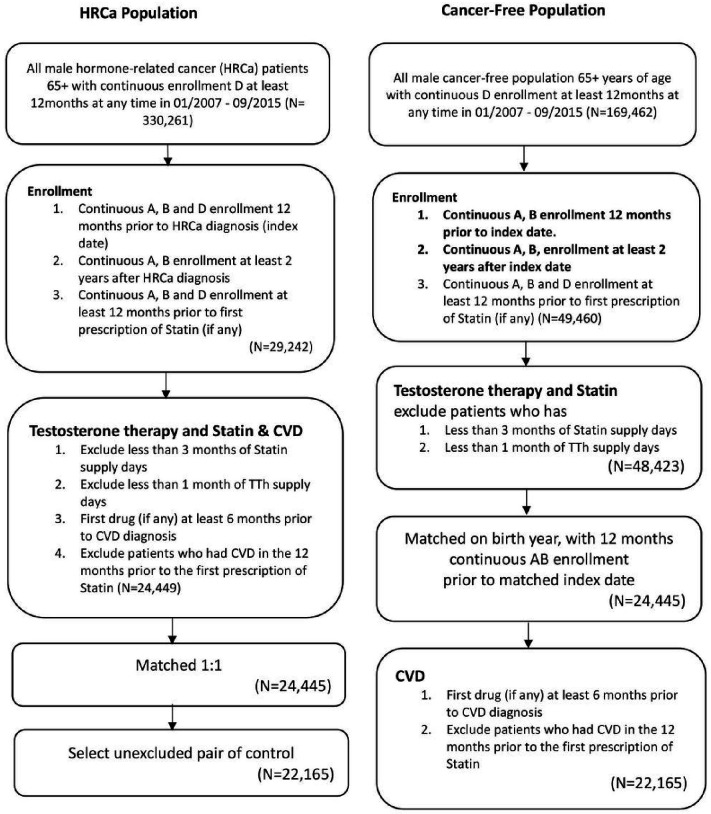
Study inclusion and exclusion criteria for identifying men with CVD development from Seer-Medicare files

The cancer cohort included all male patients aged ≥ 66 years with confirmed a primary diagnosis of prostate, colorectal, or male breast cancers (HRCa) between January 2008 and September 2015. Potential cancer cases were included if they had one-year continuous enrollment in part A, B and D prior to diagnosis of a HRCa and if they had 2 years continuous enrollment in part A and B after diagnosis of HRCa (index date, Fig. 1) as a confirmation that these patients were alive. We excluded participants who took statins but did not have at least 12 months continuous enrollment in part A, B and D prior to first prescription of statins. The patients who had less than three months’ supply of statins or patients who had less than one month supply of TTh were excluded. We further excluded patients who took statins or TTh within less than 6 months from the first diagnosis of CVD or the patients who had CVD in the 12 months before the first prescription of statins (Fig. 1).

Cancer-free cohort included male individuals aged 65 + years, matched by same birth year with cancer cohort (HRCa), and the index date was the date of diagnosis of cancer of their match. For each cancer patient, we randomly selected one cancer free-patient that fulfills the enrollment criteria based on the matched index date. In both cohorts, persons were included if they had one-year continuous enrollment in part A, B and D prior to index date (HRCa diagnosis), and if they had continuous enrollment for 2 years in Part A, and B after index date (Fig. 1). Similar exclusions and restrictions, as mentioned for HRCa participants in relation to statins and TTh prescription and diagnosis of CVD, were applied for cancer-free cohort. If a HRCa patient matched to more than one participant of the cancer-free pool only one was selected at random (Fig. 1).

### Pre-diagnostic use of testosterone replacement therapy (TTh) and Statin prescription prior to CVD development

Prescription of TTh and use of statins was identified before CVD diagnosis and obtained from Medicare Part D using National Drug Codes (NDC) and Current Procedural Terminology (CPT) codes. The primary exposures were statins (Yes/No), long term use of statins, TTh (Yes/No), months of TTh use and number of TTh injections. We categorized statins in five groups based on taking time: No use (reference group), 3–12 months, 1–4 years, 5–8 years, > 8 years. Similarly, TTh users were categorized in five groups based on time of use: No TTh (reference group), 1–6 months, 6–12 months, 12–36 months, > 36 months. For number of TTh injections, the five groups were created: No TTh (reference group), 1–2, 3–5, 6–12, > 12. For patients who took both TTh (Yes/No) or statin (Yes/No), we categorized individuals in four groups: No TTh plus No statin (reference group), statins alone, TTh alone, and TTh plus statins. For individuals who took both TTh or statins, at least 6 months between the later of the two dates and CVD diagnosis (if any was required).

### Identification of patients with cardiovascular disease (CVD)

The composite primary outcome of this study was CVD (yes/no). Individuals were classified as having CVD if they had at least one inpatient diagnosis or two or more outpatient diagnoses at least 30 days apart from one year before index date and September 2015. We excluded prevalent CVD (2007–2008) from this study, which was considered from patients with CVD in the 12 months prior for the first Statin prescription. CVD was identified by using the International Classification of Diseases (ICD) 9th revision and 10th revision codes included ischemic heart disease (ICD-9 410–414, V45.81/82, 00.66, 36; ICD-10 I20–25), stroke (ICD-9 430–435, 00.61–00.65, 38.1, 39.74; ICD-10 I60–79), and cardiomyopathy/heart failure (ICD-9 425, 428, V42.1, 37.51; ICD-10 I30-I52). Secondary outcomes were coronary heart disease (CHD) (yes/no) and stroke (yes/no), and they were also identified based on ICD-9 and ICD-10 codes (ICD-9 410–414; ICD-10 I20, I23–25).

### Covariates

Covariates included demographic, clinical, and cancer characteristics. Demographic data contained patient’s age at diagnosis, race (White, Black, Hispanic, Other), socioeconomic status including the percentage of persons older than 25 years with less than 12 years education and the percentage of adults below the poverty line in the census tract. Clinical indicators identified using NDC and CPT codes included hyperlipidemia, hypogonadism, hypertension, diabetes, use of insulin, muscular wasting and disuse atrophy, malaise and fatigue, osteoporosis, erectile dysfunction, depressive disorder, anterior pituitary disorder. In addition, we measured number of primary care physician (PCP) visits and 12 months baseline Charlson comorbidity index scores to determine comorbidity burden. Cancer characteristics included cancer stage categorized as localized (AJCC stage I and II) and advanced (AJCC stage III and IV), grade (Low and High), radiation therapy (Yes/No) and number of prostate-specific antigen (PSA) tests, number of breast cancer screening and number of colorectal cancer colonoscopy.

### Statistical Analysis

Demographic, clinical and cancer characteristics were compared by four different drug groups using Chi-square test for categorical variables and F-test for continuous variables. Cancer-free population were a 5% random sample from the Medicare population, whereas cancer patients account for 100% of the cancer population from SEER. The weighted multivariable logistic regression model was used to identify independent associations of TTh and statins with incident CVD, CHD and stroke. Using a *prioiri knowledge* of confounders, we adjusted for age at diagnosis, race, PCP visits, PSA tests, number of breast cancer screening and number of colorectal cancer colonoscopy, CCI, poverty, hyperlipidemia, hypogonadism, hypertension, diabetes, use of insulin, muscular wasting and disuse atrophy, malaise and fatigue, osteoporosis, erectile dysfunction, depressive disorder, anterior pituitary disorder, and census tract socioeconomic status. These weighted multivariable models compared the odds of incident *CVD versus no CVD, CHD versus no CHD*, and *stroke vs no-stroke*. Multiplicative interactions terms were incorporated into the models and tested using the Wald test. The significance level was set at α = 0.05. All analyses were performed with SAS 9.4 (SAS Institute, Cary, NC, US).

## Results

We identified 44,330 men in the overall cohort (Table 1), 22,165 diagnosed with HRCa (Supplemental table 1), and 22,165 age-matched cancer-free men (Supplemental table 2) in the SEER-Medicare data 2007–2015. Mean age was 75 years old, and the median follow-up time until diagnosis of CVD or end of study was 2.5 years (2015). Table 1 shows patient characteristics by combination of statin use and TTh in the overall population. Approximately 78% of men had no TTh/no statin, 17.61% used statin alone, 2.5% used TTh alone, and 1.21% used both TTh plus statin. Compared to men with no TTh plus no statin, users of statin alone, TTh alone or their combination were less likely to be diagnosed with CVD but more likely to be younger, White, hypertensive, diabetic, reported hyperlipidemia, muscular wasting, malaise and fatigue, erectile dysfunction and higher score of CCI comorbidity, hypogonadism, osteoporosis, depressive disorder, to have higher use of insulin, but with a higher number of PCP visits, PSA tests, and marginally higher percentage of adults below poverty and mean of adults with < 12 years of education. In general, frequencies of the weighted characteristics for HRCa survivors (Supplemental table 1), and the matched cancer-free cohort (Supplemental table 2) were similar as in Table 1.

The multivariable-adjusted associations of statins use and TTh, and their combination, with CVD in the overall population is shown Table 4. Compared with no statins use, statins use was independently inversely associated with incident CVD (OR = 0.51, 95% CI, 0.46–0.55), CHD (OR = 0.88, 95% CI, 0.80–0.98), and stroke (OR = 0.59, 95% CI, 0.49–0.70). Compared with no TTh use, TTh was only independently inversely associated with CVD (OR = 0.81, 95% CI, 0.67–0.97). In combination, compared with no TTh plus no statins use, TTh plus statins use was inversely associated with CVD (OR = 0.51, 95% CI, 0.37–0.69). There was no statistical interaction between TTh and statins and its association with CVD (*P_interaction_ = 0.2268)*, with CHD (*P_interaction_ = 0.1136)* and stroke (*P_interaction_ = 0.7892)*.

Among HRCs survivors, the multivariable-adjusted associations of statins use and TTh, and their combination, with CVD is shown in Table 5. Compared with no statins use, statin use was independently inversely associated with incident CVD (OR = 0.53, 95% CI, 0.49–0.58) and stroke (OR = 0.55, 95% CI, 0.46–0.65), but not with CHD (OR = 0.96, 95% CI, 0.87–1.06). Compared with no TTh use, TTh was not significantly associated with CVD, stroke and CHD. In combination, compared with no TTh plus no statins use, TTh plus statins use was inversely associated with CVD (OR = 0.60, 95% CI, 0.44–0.81) but not with CHD (OR = 1.10, 95% CI, 0.78–1.54). No evidence of statistical interactions was identified in these associations (CVD, *P_interaction_ = 0.1048*, *CHD, P_interaction_ = 0.4549*, *or stroke, P_interaction_ = 0.9296)*.

Among the cancer-free population, the multivariable-adjusted associations of statins use and TTh, and their combination, with CVD is shown in Table 6. Compared with no statin use, statin use was independently inversely associated with incident CVD (OR = 0.50, 95% CI, 0.46–0.55), CHD (OR = 0.88, 95% CI, 0.78–0.98), and stroke (OR = 0.59, 95% CI, 0.49–0.71). Compared with no TTh use, TTh was independently inversely associated with CVD (OR = 0.80, 95% CI, 0.69–0.98), but not CHD or stroke. In combination, compared with no TTh plus no statins use, TTh plus statins use was inversely associated with CVD (OR = 0.50, 95% CI, 0.36–0.70). We found no evidence of statistical interaction between TTh and statins and its association with CVD (*P*_*interaction*_ *= 0.2637)*, CHD (*P*_*interaction*_ *= 0.08) or stroke (P*_*interaction*_ *= 0.7888)*. In general, the associations of the time of statin and TTh use, including TTh injections, were not significant and sparse.

## Discussion

Overall, we found independent and inverse associations between pre-diagnostic use of statin and TTh (prior to CVD development) with incident CVD among the overall population. The combination of TTh plus statin was inversely associated with incident CVD. Among the cancer-free population, the significance and direction of these associations remained similar. However, among HRCa survivors, the independent use of statins and combination of TTh plus statins were inversely associated with incident CVD, but not the independent use of TTh. However, among HRCa survivors, the greatest reduction effects on CVD were mainly observed with statins or in combination (TTh plus statins), but not independently with TTh. To our knowledge, this is the first epidemiological study that quantifies the association of the combination between TTh and statins with CVD among HRCa survivors and a cancer free population in Seer-Medicare 2007–2015.

### Statins and TTh in relation with prostate, colorectal or male breast cancers (HRCa) survivors

An interplay between statins, HRCa and CVD was previously reported. For instance, two meta-analysis, *Tan et al*. and *Mei et al.*, reported inverse associations between statins and aggressive PCa.^11,12^ In colorectal cancer research, an umbrella systematic review and meta-analysis concluded that the association between statins and the risk of colorectal cancer is weak (non-significant in 12 RCTs and weak in 46 observational studies). Similar findings were reported between breast cancer (female) and statins.^5^ The US Preventive Services Task Force capitalized on 19 randomized trials and reported an inverse association between statins and cardiovascular mortality, stroke, and myocardial infarction.^21^ In previous studies of testosterone research, we^28–30^ and others,^31^ who capitalized on randomized clinical trials, found an inverse associations between TTh and incident PCa, high-grade and advanced-stage PCa, albeit findings from the meta-analyses were not statistically significant. A pooled analysis of two large observational and two randomized controlled trials reported that higher levels of testosterone were associated with a lower risk of colorectal cancer in men.^8^ More recently, a 2022 large European nested-case control study concluded that there is suggestive evidence for the association between testosterone and male colon cancer development.^10^ For male breast cancer, the role of endogenous and exogenous testosterone remains unclear^9,12^, which may due, in part, to small sample size studies, including our study. In parallel, inverse associations between TTh and CVD in previous systematic reviews of observational studies and randomized controlled trials were previously reported.^24^ Therefore, in this study we hypothesized that the combination of statins and TTh can be potentially inversely associated with CVD among HRCa survivors and the matched cancer-free cohort.

### Statins and TTh associated with CVD among HRCa survivors

To our knowledge, no study has investigated the combination of TTh plus statin use in relation to CVD among HRCa patients and a cancer-free population. Our study may not seem comparable with previous studies, but it is important to note there are a number of studies that have explored the interplay between statins use and TTh and CVD among HRCa (e.g. prostate, breast, endometrial) survivors (men and women) in different settings that may provide direction to our findings. For instance, Sturgeon *et al*.^32^ found that the majority of deaths from CVD occur in patients with breast, bladder, or PCa. Meanwhile, Felix *et al*.^33^ and Soisson *et al*.^34^ reported that endometrial cancer survivors were more likely to die from CVD, and were at higher risk for various adverse long-term CVD outcomes compared with women from the general population. Two other studies conducted among women with breast cancer receiving chemotherapy^35^ or chemotherapy/radiotherapy^36^ found that statins use was associated with a lower risk of incident heart failure or lower mean increase of left-ventricular ejection fraction, respectively.

Even though little is known about the interplay between TTh and CVD among HRCa survivors, recently a 6-month double-blind randomized placebo-controlled trial found that among male cancer survivors with low-normal morning total serum testosterone, the used of TTh was associated with a significant reduction of whole-body fat mass^37^, which is a strong risk factor for CVD. On the other hand, the use of androgen deprivation therapy has been reported to increase the risk of CVD or death, but the evidence remains inconsistent.^38^

Although study may not be similar to previous studies due to different methodological settings, yet what remains to be determined is whether there is an epidemiologic and biological plausibility in the interplay between low levels of testosterone/testosterone deficiency and hypercholesterolemia, including their treatment with TTh and statins, and CVD. Recently, a US commercial claims database study of 189 491 men aged 40–69 years found that 21% of TTh users also reported statin use.^28^

Furthermore, a review article by Mokarram et al. 2017 suggested the biological mechanistic interconnection between testosterone metabolism and the mevalonate pathway, which is required for the generation of several fundamental end-products including cholesterol that is a required precursor in steroidogenesis.^39^ Yet, more clinical and epidemiological studies with larger sample sizes are warranted to confirm these findings.

### Strengths and Limitations

Our study has strengths. This study included a large number of men with incident CVD, large cohort of HRCa patients and cancer-free men, and a large enough sample to investigate men who have used both statin and TTh. Our proportion of “TTh only” users (2.7%) is consistent with a recent study that reported that 2.4% of men received TTh after PCa treatment capitalizing on the Optum Clinformatics database (n = 126,734).^40^ Our study also included follow-up data of 2.5 years, and a detailed information on patient’s exposures to TTh and statin use based on filled prescriptions and inclusion of clinically relevant comorbidities.

Our study has limitations. First, cases of stroke were limited among HRCa patients and the cancer-free population. Similarly, there was a small number of TTh users with stroke that we had limited statistical power to conduct analysis in those groups. Second, although we adjusted the multivariable analysis for hypogonadism, age, Charlson Comorbidity Index, cancer diagnosis and screening, and hyperlipidemia, we cannot rule out potential residual confounding by these factors or unknown confounders. In this study we adjusted for hyperlipidemia derived from diagnosis codes; however, SEER-Medicare does not include laboratory results (serologic or diagnostic indications) to define hypercholesterolemia from serum total cholesterol, testosterone deficiency with serum total testosterone or other occupational, environmental, nutritional and/or several lifestyle factors. Third, the odds ratios provided in these analyses were obtained from weighted conditional logistic regression models that consider incident cases of CVD and an exposure (TTh and statins) that preceded the CVD diagnosis; therefore, they are only approximations to risk ratios.^41^ Fourth, there are general limitations of retrospective analysis based on Medicare claims data. For instance, comorbidities were identified using ICD-9 and ICD-10 codes, possibly resulting in incorrect capture of medical conditions due to undercoding, upcoding or miscoding, leading to classification bias in those comorbidities at baseline. It is possible that statins and TTh users and non-users might have used statins or TTh prior to 2007; however, the earliest year of available Part D data was 2007. However, this potential inaccurate capture of information will be considered nondifferential misclassification because they were collected before the disease developed, which in general influences associations to the null (1.0). Fifth, limiting the pre‐index period to 6 months did not allow to capture full medical history; for instance, patients may have had the comorbidity of interest before the start of the pre index period. Furthermore, the eligibility criteria of continuous enrollment for 2 years in Part A and B after diagnosis of HRCa, was applied to both HRCa cases and the cancer-free cohort. This was conducted to mitigate potential confounding by indication and protopathic biases. In addition, the high (90%) five-year survival rate for HRCa has the potential to have a minimal effect on this criterion. Finally, our results may not be generalizable to patients with CVD among HRCa patients using other types of insurance, no insurance at all, or a younger population (< 65 years old).

## Conclusion

In summary, in this SEER-Medicare claims-based analysis, we found that pre-diagnostic use of statins and TTh, prior to CVD development, independent or in combination, were inversely associated with incident CVD in the overall and cancer-free populations, but among HRCa survivors it was mainly use of statins, not TTh. In fact, the greatest reduction on CVD was observed with statins use or in combination with statins, but not with TTh. Future prospective studies with larger sample sizes are needed to confirm these independent and combined associations of statins and TTh with CVD among elderly men with and without aggressive HRCa.

## Figures and Tables

**Table 1:** Baseline characteristics of men 65 + years old among the overall population (N = 44,330) by current use of testosterone replacement therapy (TTh) and/or statin in the SEER-Medicare 2007–2015

Characteristics	No TTh/No Statin	Statin alone	TTh alone	TTh + Statin	p-value
	(N = 32,775)	(N = 9,797)	(N = 1,153)	(N = 605)	
**Incident CVD**^a^, **n (%)**	8946 (27.30)	2002 (20.43)	285 (24.72)	138 (22.81)	< .0001
**Age, n (%)**					
65–70	9425 (28.76)	2305 (23.53)	357 (30.96)	161 (26.61)	< .0001
70–75	10807 (32.97)	3718 (37.95)	428 (37.12)	243 (40.17)	
75–80	6511 (19.87)	2319 (23.67)	199 (17.26)	123 (20.33)	
>=80	6032 (18.40)	1455 (14.85)	169 (14.66)	78 (12.89)	
**Race, n (%)**					
Black	3014 (9.20)	831 (8.48)	61 (5.29)	34 (5.62)	< .0001
Hispanic	1129 (3.44)	411 (4.20)	17 (1.47)	23 (3.80)	
Other	2743 (8.37)	1098 (11.21)	38 (3.30)	34 (5.62)	
White	25889 (78.99)	7457 (76.12)	1037 (89.94)	514 (84.96)	
**Hyperlipidemia, n (%)**	13739 (41.92)	7800 (79.62)	657 (56.98)	491 (81.16)	< .0001
**Hypogonadism, n (%)**	588 (1.79)	233 (2.38)	534 (46.31)	263 (43.47)	< .0001
**Hypertension, n (%)**	18293 (55.81)	7449 (76.03)	766 (66.44)	461 (76.20)	< .0001
**Muscular wasting and disuse atrophy, n (%)**	524 (1.60)	185 (1.89)	44 (3.82)	19 (3.14)	< .0001
**Malaise and fatigue, n (%)**	6103 (18.62)	2108 (21.52)	381 (33.04)	228 (37.69)	< .0001
**Osteoporosis, n (%)**	1069 (3.26)	379 (3.87)	91 (7.89)	61 (10.08)	< .0001
**Erectile dysfunction organic, n (%)**	2510 (7.66)	913 (9.32)	336 (29.14)	159 (26.28)	< .0001
**Depression disorder, n (%)**	2480 (7.57)	932 (9.51)	166 (14.40)	90 (14.88)	< .0001
**Anterior pituitary disorder, n (%)**	45 (0.14)	20 (0.20)	36 (3.12)	19 (3.14)	< .0001
**Diabetes, n (%)**	8185 (24.97)	4248 (43.36)	392 (34)	301 (49.75)	< .0001
**Charlson comorbidity, n (%)**					
0	23316 (71.14)	5433 (55.46)	709 (61.49)	282 (46.61)	< .0001
1	5434 (16.58)	2453 (25.04)	245 (21.25)	155 (25.82)	
2	2144 (6.54)	953 (9.73)	110 (9.54)	83 (13.72)	
3 or more	1881 (5.74)	958 (9.78)	89 (7.72)	85 (14.05)	
**Use of insulin, n (%)**	661 (2.02)	459 (4.69)	27 (2.34)	32 (5.29)	< .0001
**Number of PSA**^b^ **tests, mean (SD)**	0.92 (1.06)	1.18 (1.11)	1.32 (1.23)	1.51 (1.28)	< .0001
**Number of PCP visits**^c^, **mean (SD)**	14.38 (14.97)	17.62 (15.21)	22.42 (15.85)	26.51 (19.21)	< .0001
**Percent of adults with < 12 years education, mean (SD)**	19.67 (12.77)	20.60 (13.26)	17.35 (11.37)	18.67 (13.03)	< .0001
**Percent of adults below poverty, mean (SD)**	12.06 (8.81)	12.31 (8.89)	11.31 (8.08)	12.02 (8.43)	0.0021

a Cardiovascular diseases (CVD);

b Prostate specific antigen (PSA);

c Primary care physician (PCP)

**Table 4:** Overall population- independent and combined associations of TTh and statin use with incident CVD, CHD and stroke in the overall population, SEER-Medicare 2007–2015

	Incident CVD^†^			Incident CHD^†^			Incident Stroke^†^		
	Event	OR	95% CI	Event	OR	95% CI	Event	OR	95% CI
	Rate			Rate			Rate		
**Statin (YesvsNo)**	19601/ 95180	0.51	0.46, 0.55	12731/ 95180	0.88	0.80, 0.98	3310/ 95180	0.59	0.49, 0.70
**Statin, long term use**									
No use	98816/ 370285	1		40469/ 370285	1		18964/ 370285	1	
3–12 months	2819/ 18030	0.38	0.31, 0.46	1680/ 18030	0.66	0.53, 0.84	418/ 18030	0.42	0.27, 0.66
1–4 years	6595/ 38800	0.41	0.36, 0.47	4345/ 38800	0.76	0.65, 0.89	916/ 38800	0.40	0.30, 0.54
5–8 years	2396/ 10678	0.53	0.43, 0.66	1571/ 10678	0.92	0.72, 1.17	330/ 10678	0.51	0.32, 0.83
>8 years	7791/ 27672	0.72	0.63, 0.82	5135/ 27672	1.16	1.00, 1.35	1646/ 27672	0.98	0.77, 1.24
*P for trend*	< .0001			< .0001			< .0001		
**TTh (YesvsNo)**	4204/ 17718	0.81	0.67, 0.97	1924/ 17718	0.80	0.63, 1.03^a^	729/ 17718	0.90	0.60, 1.33
**TTh, number of months of use**									
No TTh	114213/ 447747	1		51276/ 447747	1		21545/ 447747	1	
1–6 months	1380/ 5900	0.78	0.59, 1.03^b^	666/ 5900	0.86	0.59, 1.25	152/ 5900	0.52	0.26, 1.06
6–12 months	534/ 2310	0.81	0.50, 1.29	196/ 2310	0.63	0.34, 1.20	125/ 2310	1.12	0.47, 2.69
12–36 months	734/ 3196	0.74	0.50, 1.10	363/ 3196	0.86	0.52, 1.42	104/ 3196	0.7	0.27, 1.82
>36 months	209/ 1498	0.45	0.21, 0.96	67/ 1498	0.40	0.13, 1.19	60/ 1498	0.96	0.28, 3.30
*P for trend*	0.0622			0.2786			0.4265		
**TTh, number of injections**									
No TTh	114213/ 447747	1		51276/ 447747	1		21545/ 447747	1	
1–2	652/ 2680	0.77	0.52, 1.14	314/ 2680	0.78	0.45, 1.35	107/ 2680	0.8	0.37, 1.75
3–5	441/ 1245	1.45	0.81, 2.58	172/ 1245	1.16	0.57, 2.38	101/ 1245	1.72	0.68, 4.38
6–12	317/ 1248	0.68	0.37, 1.25	169/ 1248	0.7	0.31, 1.56	42/ 1248	0.57	0.13, 2.49
>12	724/ 2438	1.23	0.80, 1.90	313/ 2438	1.04	0.56, 1.94	124/ 2438	1.08	0.45, 2.57
*P for trend*	0.1959			0.7789			0.6781		
**TTh & Statin Use**									
No TTh/ No Statin	91361/ 339145	1		37466/ 339145	1		17351/ 339145	1	
Statin alone	17614/ 85477	0.50	0.46, 0.54	11422/ 85477	0.87	0.78, 0.96	3058/ 85477	0.59	0.49, 0.71
TTh alone	2813/ 11402	0.79	0.64, 0.98	1024/ 11402	0.69	0.51, 0.95	559/ 11402	0.96	0.62, 1.49
TTh + Statin	1197/ 5245	0.51	0.37, 0.69	795/ 5245	0.92	0.64, 1.32	168/ 5245	0.51	0.25, 1.04^c^

Interaction term for TTh and statin use and its association with incident CVD: *P*_*interaction*_*=* 0.2268, incident CHD: *P*_*interaction*_*=* 0.1136, Incident Stroke: *P*_*interaction*_*=* 0.7892

† Multivariable analysis adjusted for age, race, Charlson Comorbidity Index (CCI), hyperlipidemia, hypogonadism, hypertension, diabetes, use of insulin, muscular wasting and disuse atrophy, malaise and fatigue, osteoporosis, erectile dysfunction, depressive disorder, anterior pituitary disorder, percentage of adults below poverty line at census tract level, patients’ primary care (PCP), prostate-specific antigen (PSA), hormone related cancer (yes or no), Breast cancer screening, colorectal cancer colonoscopy in the conditional logistics regression with weight.

Pvalues of borderline associations:

a. 0.08;

b. 0.083;

c. 0.06

SEER-Medicare guideline presentation has been followed and all counts less than 11 have been suppressed.

**Table 5:** Independent and combined associations of TTh and statin use with incident CVD, CHD and stroke among HRCa survivors, SEER-Medicare 2007–2015

	Incident CVD^†^			Incident CHD^†^			Incident Stroke^†^		
	Event	OR	95% CI	Event	OR	95% CI	Event	OR	95% CI
	Rate			Rate			Rate		
**Statin (Yes vs No)**	1221/ 5940	0.53	0.49, 0.58	831/ 5940	0.96	0.87, 1.06	170/ 5940	0.55	0.46, 0.65
**Statin, long term use**									
No use	4516/ 16225	1		1849/ 16225	1		744/ 16225	1	
3–12 months	159/ 990	0.41	0.34, 0.50	100/ 990	0.73	0.58, 0.91	18/ 990	0.35	0.22, 0.57
1–4 years	375/ 2440	0.39	0.34, 0.44	245/ 2440	0.70	0.60, 0.81	56/ 2440	0.47	0.36, 0.62
5–8 years	156/ 678	0.58	0.48, 0.71	111/ 678	1.10	0.89, 1.37	30/ 678	0.81	0.55, 1.19
>8 years	531/ 1832	0.80	0.71, 0.90	375/ 1832	1.40	1.22, 1.60	66/ 1832	0.65	0.49, 0.84
*P for trend*	< .0001			< .0001			< .0001		
**TTh (Yes vs No)**	224/ 918	0.89	0.73, 1.09	124/ 918	1.05	0.83, 1.33	29/ 918	0.72	0.46, 1.14
**TTh, number of months of use**									
No TTh	5513/ 21247	1		2556/ 21247	1		885/ 21247	1	
1–6 months	80/ 360	0.79	0.60, 1.04	46/ 360	0.97	0.70, 1.36	12/ 360	0.79	0.44, 1.42
6–12 months	34/ 130	0.98	0.61, 1.56	16/ 130	0.97	0.53, 1.75	< 11/ 130	0.71	0.24, 2.11
12–36 months	34/ 136	1.01	0.61, 1.67	23/ 136	1.46	0.83, 2.57	< 11/ 136	0.68	0.23, 1.98
>36 months	< 11/ 58	0.55	0.28, 1.10	< 11/ 58	0.99	0.45, 2.18	< 11/ 58	N/A	N/A
*P for trend*	0.2505			0.7604			< .0001		
**TTh, number of injections**									
No TTh	5513/ 21247	1		2556/ 21247	1		885/ 21247	1	
1–2	32/ 140	0.75	0.47, 1.20	14/ 140	0.75	0.41, 1.41	< 11/ 140	1.21	0.54, 2.74
3–5	21/ 65	1.40	0.78, 2.53	12/ 65	1.60	0.83, 3.10	< 11/ 65	0.35	0.05, 2.52
6–12	17/ 68	0.81	0.42, 1.55	< 11/ 68	0.92	0.43, 1.97	< 11/ 68	0.31	0.04, 2.23
>12	24/ 98	0.72	0.40, 1.30	13/ 98	0.96	0.48, 1.92	< 11/ 98	0.97	0.32, 2.93
*P for trend*	0.3691			0.5780			0.6088		
**TTh & Statin Use**									
No TTh/ No Statin	4371/ 15649	1		1781/ 15649	1		732/ 15649	1	
Statin alone	1142/ 5598	0.52	0.48, 0.57	775/ 5598	0.95	0.86, 1.05	162/ 5598	0.55	0.46, 0.66
TTh alone	145/ 576	0.85	0.67, 1.07	68/ 576	0.98	0.73, 1.32	21/ 576	0.75	0.45, 1.26
TTh + Statin	79/ 342	0.60	0.44, 0.81	56/ 342	1.10	0.78, 1.54	< 11/ 342	0.43	0.20, 0.92

Interaction term for TTh and statin use and its association with Incident CVD: *P*_*interaction*_ *=* 0.1048, Incident CHD: *P*_*interaction*_ *=* 0.4549, Incident Stroke: *P*_*interaction*_ *=* 0.9296

† Multivariable analysis adjusted for age, race, Charlson Comorbidity Index (CCI), hyperlipidemia, hypogonadism, hypertension, diabetes, use of insulin, muscular wasting and disuse atrophy, malaise and fatigue, osteoporosis, erectile dysfunction, depressive disorder, anterior pituitary disorder, percentage of adults below poverty line at census tract level, patients’ primary care (PCP), prostate-specific antigen (PSA), hormone related cancer (yes or no), Brest cancer screening, colorectal cancer colonoscopy in the conditional logistics regression with weight.

SEER-Medicare guideline presentation has been followed and all counts less than 11 have been suppressed.

**Table 6:** Independent and combined associations of TTh and statin use with incident CVD, CHD and stroke among the cancer-free population, SEER-Medicare 2007–2015

	Incident CVD^†^			Incident CHD^†^			Incident Stroke^†^		
	Event	OR	95% CI	Event	OR	95% CI	Event	OR	95% CI
	Rate			Rate			Rate		
**Statin (Yes vs No)**	18380/ 89240	0.50	0.46, 0.55	11900/ 89240	0.88	0.78, 0.98	18220/ 89240	0.59	0.49, 0.71
**Statin, long term use**									
No use	94300/ 354060	1		38620/ 354060	1		18220/ 354060	1	
3–12 months	2660/ 17040	0.38	0.31, 0.46	1580/ 17040	0.66	0.51, 0.84	400/ 17040	0.43	0.27, 0.67
1–4 years	6220/ 36360	0.41	0.36, 0.48	4100/ 36360	0.77	0.65, 0.91	860/ 36360	0.39	0.28, 0.55
5–8 years	2240/ 10000	0.53	0.42, 0.67	1460/ 10000	0.91	0.69, 1.19	300/ 10000	0.49	0.29, 0.83
>8 years	2240/ 25840	0.72	0.62, 0.83	4760/ 25840	1.15	0.97, 1.35	1580/ 25840	1.00	0.78, 1.28
*P for trend*	< .0001			< .0001			< .0001		
**TTh (Yes vs No)	3980/ 16800	0.80	0.65, 0.98	1800/ 16800	0.79	0.61, 1.03	700/ 16800	0.90	0.59, 1.38
**TTh, number of months of use									
No TTh	108700/ 426500	1		48720/ 426500	1		20660/ 426500	1	
1–6 months	1300/ 5540	0.78	0.57, 1.06	620/ 5540	0.85	0.56, 1.29	140/ 5540	0.51	0.24, 1.09
6–12 months	500/ 2180	0.79	0.48, 1.32	180/ 2180	0.61	0.31, 1.22	120/ 2180	1.14	0.46, 2.83
12–36 months	700/ 3060	0.73	0.48, 1.11	340/ 3060	0.84	0.49, 1.43	100/ 3060	0.71	0.26, 1.90
>36 months	200/ 1440	0.45	0.20, 0.99	60/ 1440	0.37	0.11, 1.26	60/ 1440	1.00	0.29, 3.49
*P for trend*	0.0878			0.3006			0.4607		
**TTh, number of injections									
No TTh	108700/ 426500	1		48720/ 426500	1		20660/ 426500	1	
1–2	620/ 5540	0.77	0.50, 1.19	300/ 5540	0.78	0.44, 1.39	100/ 5540	0.79	0.31, 1.98
3–5	420/ 2180	1.45	0.79, 2.68	160/ 2180	1.14	0.53, 2.46	100/ 2180	1.79	0.69, 4.65
6–12	300/ 3060	0.67	0.35, 1.30	160/ 3060	0.69	0.29, 1.63	40/ 3060	0.58	0.13, 2.66
>12	700/ 1440	1.26	0.79, 2.00	300/ 1440	1.04	0.54, 2.02	120/ 1440	1.08	0.44, 2.69
*P for trend*	0.2417			0.8116			0.6696		
**TTh & Statin Use									
No TTh/ No Statin	91500/ 342520	1		37620/ 342520	1		17680/ 342520	1	
Statin alone	17200/ 83980	0.50	0.45, 0.55	11100/ 83980	0.86	0.77, 0.97	2980/ 83980	0.59	0.49, 0.72
TTh alone	2800/ 11540	0.79	0.63, 1.00	1000/ 11540	0.68	0.49, 0.95	540/ 11540	0.97	0.61, 1.55
TTh + Statin	1180/ 5260	0.50	0.36, 0.70	800/ 5260	0.91	0.62, 1.34	160/ 5260	0.52	0.24, 1.09

Interaction term for TTh and statin use and its association with Incident CVD: *P*_*interaction*_ *=* 0.2637, Incident CHD: *P*_*interaction*_ *=* 0.0800, Incident Stroke: *P*_*interaction*_ *=* 0.7888

† Multivariable analysis adjusted for age, race, Charlson Comorbidity Index (CCI), hyperlipidemia, hypogonadism, hypertension, diabetes, use of insulin, muscular wasting and disuse atrophy, malaise and fatigue, osteoporosis, erectile dysfunction, depressive disorder, anterior pituitary disorder, percentage of adults below poverty line at census tract level, patients’ primary care (PCP), prostate-specific antigen (PSA), hormone related cancer (yes or no), Brest cancer screening, colorectal cancer colonoscopy in the conditional logistics regression with weight.

SEER-Medicare guideline presentation has been followed and all counts less than 11 have been suppressed.

## Data Availability

The authors confirm that data used in developing this article are available upon reasonable request to the corresponding author.
